# Micro-Raman Characterization of Structural Features of High-k Stack Layer of SOI Nanowire Chip, Designed to Detect Circular RNA Associated with the Development of Glioma

**DOI:** 10.3390/molecules26123715

**Published:** 2021-06-18

**Authors:** Yuri D. Ivanov, Kristina A. Malsagova, Vladimir P. Popov, Igor N. Kupriyanov, Tatyana O. Pleshakova, Rafael A. Galiullin, Vadim S. Ziborov, Alexander Yu. Dolgoborodov, Oleg F. Petrov, Andrey V. Miakonkikh, Konstantin V. Rudenko, Alexander V. Glukhov, Alexander Yu. Smirnov, Dmitry Yu. Usachev, Olga A. Gadzhieva, Boris A. Bashiryan, Vadim N. Shimansky, Dmitry V. Enikeev, Natalia V. Potoldykova, Alexander I. Archakov

**Affiliations:** 1 Laboratory of Nanobiotechnology, Institute of Biomedical Chemistry, 119121 Moscow, Russia; yurii.ivanov.nata@gmail.com (Y.D.I.); t.pleshakova1@gmail.com (T.O.P.); rafael.anvarovich@gmail.com (R.A.G.); ziborov.vs@yandex.ru (V.S.Z.); alexander.archakov@ibmc.msk.ru (A.I.A.); 2 Rzhanov Institute of Semiconductor Physics, Siberian Branch of Russian Academy of Sciences, 630090 Novosibirsk, Russia; popov@isc.nsc.ru; 3 Laboratory of Experimental Mineralogy and Crystallogenesis, Sobolev Institute of Geology and Mineralogy, Siberian Branch of Russian Academy of Sciences, 630090 Novosibirsk, Russia; spectra@igm.nsc.ru; 4 Joint Institute for High Temperatures of Russian Academy of Sciences, 125412 Moscow, Russia; aldol@ihed.ras.ru (A.Y.D.); ofpetrov@ihed.ras.ru (O.F.P.); 5 K. A. Valiev Institute of Physics and Technology of the Russian Academy of Sciences, 117218 Moscow, Russia; miakonkikh@ftian.ru (A.V.M.); rudenko@ftian.ru (K.V.R.); 6 JSC Novosibirsk Plant of Semiconductor Devices with OKB, 630082 Novosibirsk, Russia; gluhov@nzpp.ru; 7 Russian Union of Industrialists and Entrepreneurs, 109240 Moscow, Russia; arhsmirnov@mail.ru; 8 Federal State Autonomous Institution “N. N. Burdenko National Medical Research Center of Neurosurgery” of the Ministry of Health of the Russian Federation, 125047 Moscow, Russia; dousachev@nsi.ru (D.Y.U.); ogadjieva@nsi.ru (O.A.G.); bbashiryan@nsi.ru (B.A.B.); Shimava@nsi.ru (V.N.S.); 9 Institute for Urology and Reproductive Health, Sechenov University, 119992 Moscow, Russia; enikeev_dv@mail.ru (D.V.E.); potoldykovanv@gmail.com (N.V.P.)

**Keywords:** nanowire chip, SOI, circular RNA, circ-SHKBP1, micro-Raman spectroscopy, high-k stack layer

## Abstract

The application of micro-Raman spectroscopy was used for characterization of structural features of the high-k stack (h-k) layer of “silicon-on-insulator” (SOI) nanowire (NW) chip (h-k-SOI-NW chip), including Al_2_O_3_ and HfO_2_ in various combinations after heat treatment from 425 to 1000 °C. After that, the NW structures h-k-SOI-NW chip was created using gas plasma etching optical lithography. The stability of the signals from the monocrine phase of HfO_2_ was shown. Significant differences were found in the elastic stresses of the silicon layers for very thick (>200 nm) Al_2_O_3_ layers. In the UV spectra of SOI layers of a silicon substrate with HfO_2_, shoulders in the Raman spectrum were observed at 480–490 cm^−1^ of single-phonon scattering. The h-k-SOI-NW chip created in this way has been used for the detection of DNA-oligonucleotide sequences (oDNA), that became a synthetic analog of circular RNA–circ-SHKBP1 associated with the development of glioma at a concentration of 1.1 × 10^−16^ M. The possibility of using such h-k-SOI NW chips for the detection of circ-SHKBP1 in blood plasma of patients diagnosed with neoplasm of uncertain nature of the brain and central nervous system was shown.

## 1. Introduction

**Figure 1:**
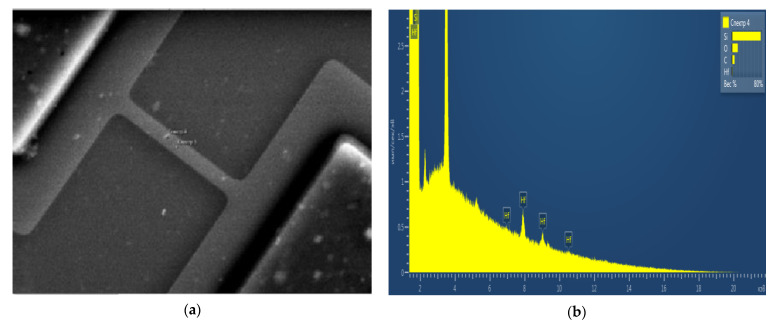
(**a**) SEM microimage of a nanotape transistor (W × L = 1.2 × 14 µm^2^) after measurements without PMA heat treatment; (**b**) EDX spectrum at point 4 (flake) showing the presence of hafnium on the silicon surface.

Raman spectroscopy can be applied in different forms of research, including for the development of biosensors [1,2]. Concurrently, Raman spectroscopy application in the development of highly sensitive nanotechnology-based diagnostic devices is propitious [2,3]. As the concentration of biomolecules in blood at an early stage of cancer pathology can be at the level of 10^−15^ M or lower [4], there must be very high concentration sensitivity recognition. For this reason, novel techniques for the diagnosis of an early-stage cancer are required. Therefore, the development of molecular detectors providing an opportunity to recognise biological macromolecules at the single molecule level, without requiring amplification and free from the previously mentioned drawbacks, is a prospective direction of research. A biosensor based on nanowire structures “silicon-on-insulator” (NW biosensor) can be considered an example of such a molecular detector [5,6,7]. They perform real time label-free detection of biological macromolecules with high sensitivity (at femtomolar and even sub-femtomolar levels) and thus, set forth a promising basis for the development of novel analytical systems. The modulation registration of current flowing through the NW structure upon adsorption of analyte molecules onto its surface provides the basis for the NW biosensor operation principle [8,9,10,11]. High surface-to-volume ratio determines the high sensitivity of the NW biosensor [12], and defines the development of chips with the smallest dimensions of sensor elements to be a relevant task. Theoretical detection limits achievable with such a biosensor can reach the level of single molecules [13]. To date, however, there are problems related to the stability of the signal from the NW structure when analysing liquids, including multicomponent matrix (plasma or serum). This is especially evident in the presence of only natural oxide with a thickness of less than 2 nm on the silicon surface [14]. In the study [14], high dielectric constant (high-k) hafnium dioxide HfO_2_ was proposed to be employed as a protective oxide. The dioxide should be deposited by the plasma-enhanced atomic layer deposition (PEALD) method, followed by post-deposition annealing (PDA) in forming gas in order to not reduce the sensitivity of the sensors. However, experiments with such coatings showed their instability in biofluids, and coating delamination was detected (Figure 1). After measuring, the energy-dispersive X-ray spectroscopy (EDX) signal on the chip surface from hafnium is observed only on micro flakes in a scanning electron microscope (SEM). Micro-Raman spectroscopy is a faster method to control the quality of coatings and the production of such chips.

High sensitivity and stability of nanowire sensors with a PEALD coating with aluminum oxide after high-temperature annealing in biofluids has already been demonstrated [15]. However, the change in the properties of silicon nanowires after heat treatment of materials with strongly different thermal expansion coefficients, required to reduce the density of electronic states at the interface of the protective dielectric with silicon and to create ohmic contacts during postmetallization annealing (PMA), remained unclear. To overcome this problem, the changes used micro-Raman spectra from nanowires with coatings of aluminum and hafnium oxides.

In the present study, micro-Raman spectroscopy has been employed for monitoring the formation processes of the chip surface к NW biosensor. Micro-Raman spectroscopy has been implemented to observe and check the formation processes of the h-k-SOI-NW chip surface with high-k coating (h-k-SOI-NW chip), which was later used for the detection of oDNA, i.e., a synthetic analogue of circ-SHKBP1, which correlates with the development of human glioma [16]. 

Glioma is one of the most common types of primary brain tumor, difficult to be diagnosed and has an unfavorable prognosis [17,18]. Methods for diagnosing glioma include liquid biopsy [19,20] and identification of serological biomarkers [21,22]. However, low sensitivity is among the disadvantages of these methods [4,23]. Thus, protein biomarkers such as OPN, CEA, galectin-1, PDGF, IGFBP-2, MMP-9, and YKL-40, which employ enzyme-linked immunosorbent assays (ELISA) [24], are considered molecular markers associated with the development of glioma. The sensitivity of the analysis is ~10^−12^ M [25]. Nucleic acids, for example, microRNA, circulating DNA and RNA, the concentration of which is determined by polymerase chain reaction (PCR), can also be used as markers associated with the development of glioma [24]. This method is highly sensitive and specific. A small amount of biomaterial is required for the analysis [26]. However, using labels to increase the sensitivity of the method, as well as the high probability of causing sample contamination, can often lead to false positive results.

Since the therapy of oncological diseases is more effective at an early stage, the development of new highly sensitive methods to detect oncological markers is urgent. Such methods can include nanowire systems based on field nanotransistor chips, in which a chip surface is covered with a high-k stack layer (h-k-SOI-NW chip) to ensure staging.

Circular RNA is a type of RNA molecule whose ends are locked by valence bonds. Circular RNAs can be formed from introns or as a result of loops appearing in various parts of the maturing transcript. The specific functions of circular RNAs are not fully understood, but there is high probability that they are involved in the regulation of gene expression [27,28,29]. Circular RNAs are fairly stable structures and due to their resistance to exonuclease degradation, circulating RNAs can be accumulated in cells [30]. About one thousand circulating RNAs were identified in human serum [31]. 

Moreover, there are more circulating RNAs in peripheral blood than in organ cells. The reasons for this are not clear, but circular RNAs can be used as markers for determining the stages of cancer, as well as other diseases, such as coronary heart disease, diabetes, and multiple sclerosis [32]. 

It was shown that such a h-k-SOI-NW chip with immobilized oDNA analogues of circ-SHKBP1–probes can be effectively employed in the detection of complementary oDNAs with high concentration sensitivity (detection limit (DL) ~10^−16^ M) and in the detection of circRNA in blood plasma patients diagnosed with neoplasm of uncertain nature of the brain and central nervous system. In the future, such a chip can be used to create a new highly sensitive glioma diagnosticum.

## 2. Results

Earlier in our study [14], the usage of high-permittivity (high-k) HfO_2_ as a protective coating applied by the PEALD method followed by PDA in forming gas was proposed. It aimed to enhance the stability of the sensor sensitivity. However, experiments with the use of such coatings have shown their instability in bioliquids, accompanied by the separation of coatings. Therefore, the method of quality control for the production of such chips using micro-Raman spectroscopy was employed.

### 2.1. Monitoring of the Quality of h-k-SOI-NW Chip with High-k Cover Dielectrics of Different Composition by Micro-Raman Spectroscopy

**Figure 2:**
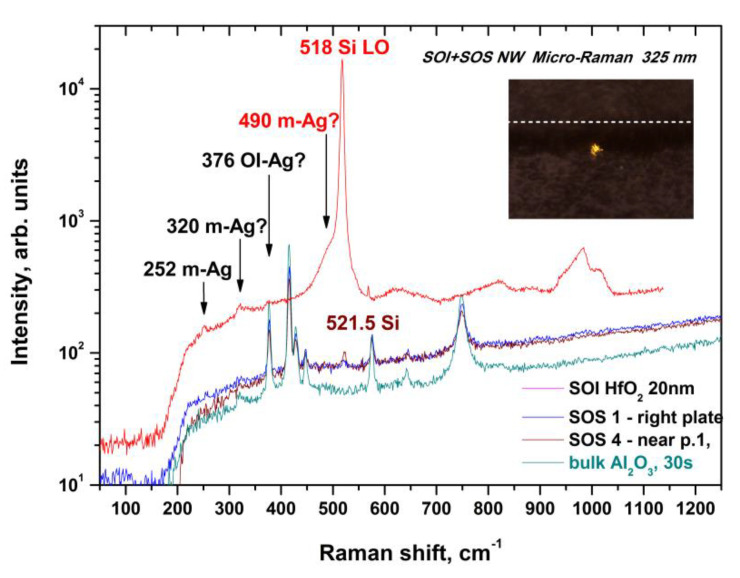
Micro-Raman spectra taken in the direction of the ultraviolet (UV) laser beam across the silicon surface along the [110] axis for SOI (upper spectrum) and SOS (silicon-on-sapphire, lower spectra) nanowire transistors with W × L = 1.2 × 14 µm^2^, after annealing at 1100 °C and a measurement cycle. The inset shows a micrograph of the measurement geometry of two identical halves (SOS1 and SOS4) of the chip, folded with the front side of the nanowires to each other and a UV laser spot with a diameter of 2 microns mainly on the lower half (SOS4) of the SOS chip, as well as a UV laser spot at a distance of 50 microns from its surface (bulk Al_2_O_3_). Arrows indicate spectrum lines corresponding to monoclinic (m) and orthorhombic (OI) phase phonons according to [14,33]. The “?” symbol means that there is no reliable information about the nature of the lines marked, except theoretical estimates, and they possibly overlap with the lines of Raman scattering of silicon dioxide from the layer of a buried dielectric SOI structure.

Quality monitoring of the h-k-SOI-NW chip with high-k cover dielectrics Al_2_O_3_/HfO_2_; Al_2_O_3_/HfO_2_/Al_2_O_3_ was conducted using micro-Raman spectroscopy. The spectra were sensitized by laser radiation in the UV (325 nm) range. Figure 2 shows examples of the micro-Raman spectra obtained in this way.

The micro-Raman spectroscopy with the UV excitation shows that hafnium dioxide is mainly in the non-polar monoclinic phase, while the «silicon-on-sapphire» (SOS) and SOI structures of the chips differ markedly after annealing with RS signals from the silicon layer (Figure 2). If the SOI RS signal of single-phonon scattering on LO phonons of silicon (520.5 cm^−1^) is shifted towards smaller wave numbers, which indicates tensile stresses, then the SOS is shifted to the right, which means that the compression stresses in the silicon film are up to 0.5%. 

**Figure 3:**
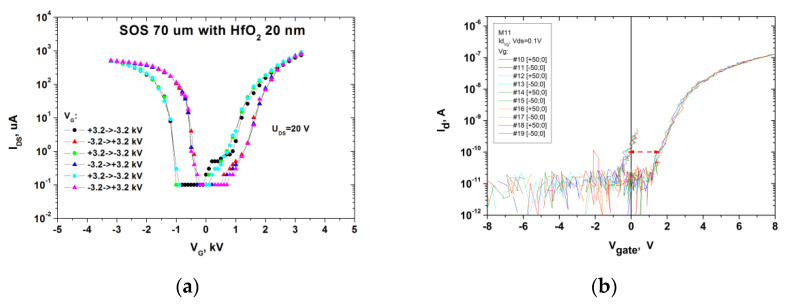
(**a**) Drain-gate characteristics of SOS transistors with a 20 nm layer of HfO_2_ nanowire transistors with W × L = 3 × 10 µm^2^, after annealing at 1000 °C and 3 measurement cycles with a higher steepness of the p-channel transistor; (**b**) drain-gate characteristics in SOI nanowire transistors of the n-type with h-k stack HfO_2_ (8 nm)/Al_2_O_3_ (2 nm)/NW Si (30 nm) from 5 scan cycles.

It is known that such compression stresses lead to a 20% increase in the mobility of holes and an increase in the steepness of the gate characteristics in the p-channel transistor, and tension stresses in the n-channel transistor with corresponding increase in sensitivity. Indeed, this behaviour of the sub-threshold slope is actually observed for the SOS of p-type nanotransistors and the SOI of n-type nanotransistors (Figure 3).

The stability of the transistor characteristics was tested on hundreds of scan cycles. The 600 V memory windows on the HfO_2_ (20 nm) SOS and 1.5 V on the Al_2_O_3_ (2 nm)/HfO_2_ (8 nm) SOI are stable and associated with the ferroelectric orthorhombic OI phase of hafnium dioxide. At the same time, for structures with an Al_2_O_3_ (2 nm)/HfO_2_ (6 nm)/Al_2_O_3_ (2 nm) dielectric, after PMA heat treatments such hysteresis is absent, possibly due to amorphous aluminum oxide layers crystallising at temperatures > 1000 °C. Probably, the recrystallisation of HfO_2_ in the structure of the orthorhombic phase (OI line 376 cm^−1^ in Figure 2) requires contact with a single-crystal substrate during annealing. Unfortunately, on sapphire, this line overlaps with one of the substrate lines. 

An increase in the thickness of the hafnium dioxide layer increases both the RS peak of 320 cm^−1^ from the monoclinic phase and the shoulder (486 cm^−1^), which was observed earlier on a stretched layer of “disturbed” silicon (Figure 2 and Figure 3) [34]. 

**Figure 4:**
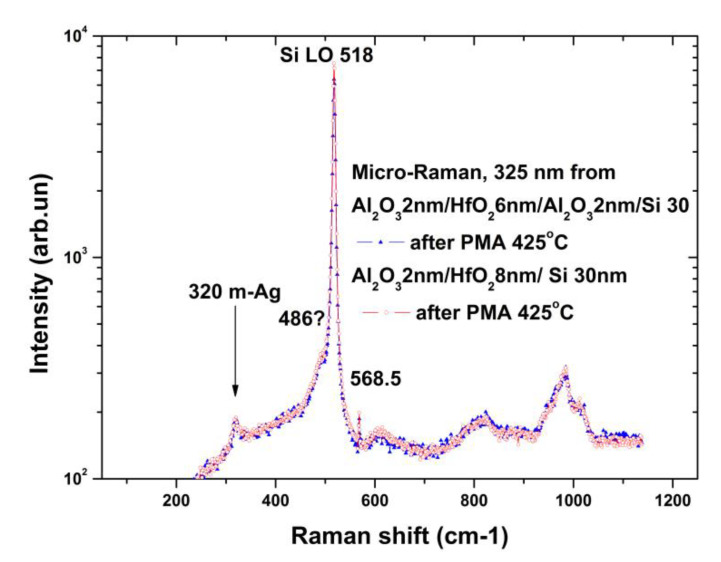
UV micro-Raman spectra for NW chips on a SOITEC h-k stack Al_2_O_3_ (2 nm)/HfO_2_ (6 nm)/Al_2_O_3_ (2 nm) and on a SOI plate (ISP SB RAS) with an h-k stack HfO_2_ (8 nm)/Al_2_O_3_ (2 nm). Arrows indicate spectrum lines corresponding to monoclinic (m) phase phonons according to [14,33]. The “?” symbol means that there is no reliable information about the nature of the lines marked, except theoretical estimates, and they possibly overlap with the lines of Raman scattering of silicon dioxide from the layer of a buried dielectric SOI structure.

However, our measurements on the p-type chip with a SOITEC SOI wafer with the edge clipping (i.e., on a substrate), and between chips in the cutting area on SOI wafer produced by ISP SB RAS (i.e., on a substrate), where disturbances might result from chemical etching, also show the same shoulder (Figure 4).

Disturbances in the 30 nm thick SOI layer are difficult to distinguish due to the noticeable contribution of the signal from the Si layer and the possible overlap with the 490 cm^−1^ line from the monoclinic phase of hafnium dioxide (Figure 2). The peak of 568.5 cm^−1^ observed in the spectra is an artefact (Figure 4).

### 2.2. Demonstration of the Detection Capabilities of Synthetic Analogues of circRNA-oDNA, Associated with Glioma by h-k-SOI-NW Chip

**Figure 5:**
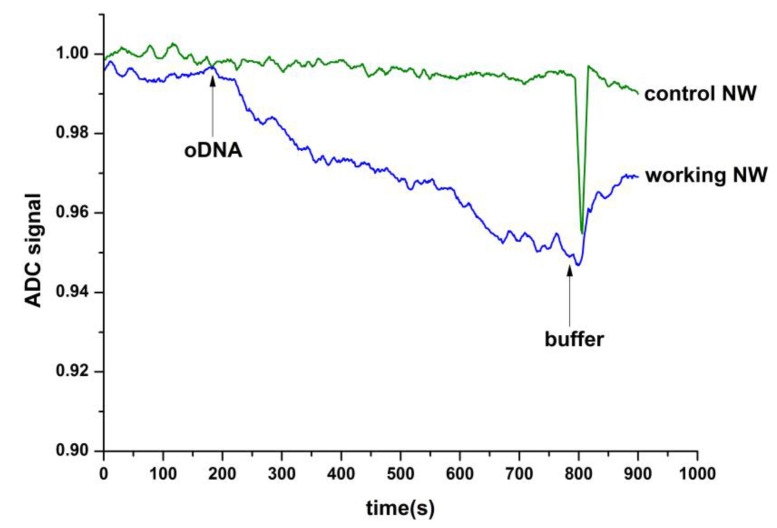
The results acquired in the detection process of target oDNA in buffer using an h-k-SOI-NW chip with n-type conductance. Typical sensogram curves were acquired upon the analysis of solutions with oDNA (analogue of circ-SHKBP1) concentration 1.1 × 10^−16^ M (blue curve) and buffer solutions without oDNA. Conditions of the experiment: control NW—sensor without immobilized oDNA probes (green curve); working NW—sensor sensitized with covalently immobilized oDNA probes (blue curve); 1 mM potassium phosphate buffer (pH 7.4); V_g_ = 41 V; V_ds_ = 0.1 V. Arrows indicate the addition of analysing solution and the wash with pure potassium phosphate buffer.

It was shown that the manufactured h-k-SOI-NW chip can be employed to detect synthetic oDNA, which is the analogue of circ-SHKBP1. For this purpose, the NW sensors of an h-k-SOI-NW chip were modified with oDNA probes complementary to the targets, according to the Materials and Methods section. Solution of target oDNA was analysed at a very low concentration in the sub-femtomolar range ~10^−16^ M, which is regarded as a distinctive amount of the concentration of biomarkers at the early stage of oncological and contagious diseases [4]. To control non-specific adsorption, we used additional NW sensors, located on the same chip, not sensitized with any oDNA. The resulting detection signal of target oDNA in buffer is demonstrated in Figure 5.

It could be inferred from this figure that when analysing the target oDNA solution, there is a decrease in the signal from the NW sensor. At the same time, for control NW without target oDNA, there is no change in the signal level. This indicates the presence of a biospecific binding between sensor-immobilized oDNA samples and the target oDNA molecules from the analysed solution. Thus, the created h-k-SOI-NW chip allows detection of oDNAs associated with the development of glioma with femtomolar sensitivity.

### 2.3. Demonstration of the Detection Capabilities of circ-SHKBP1, Associated with Glioma in Blood Plasma by h-k-SOI-NW Chip

**Figure 6:**
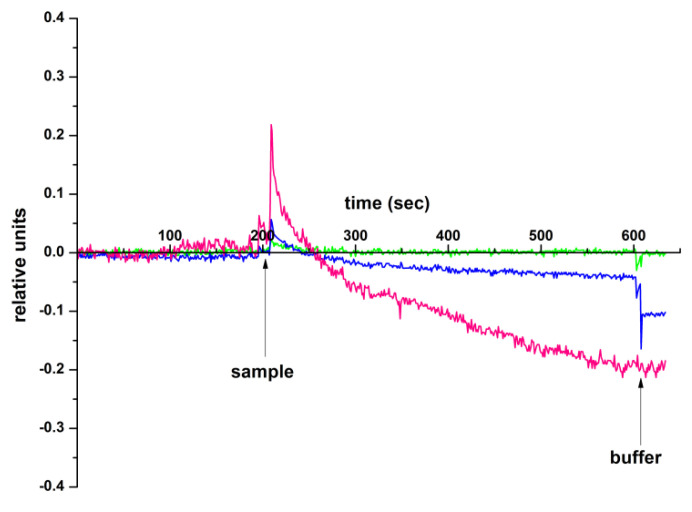
Difference signal obtained with the use of the oDNA-sensitized h-k-SOI-NW chip with n-type conductance, obtained upon the purified buffer examination (control) (green line), upon the analysis of circRNA isolated from plasma of a patient with a prostatic hyperplasia (blue line), and upon the analysis of circRNA isolated from plasma of a neoplasm of uncertain nature of the brain and central nervous system patient (pink line). Experimental conditions: 1 mM potassium phosphate buffer, pH 7.4; V_g_ = 41 V; V_ds_ = 0.1 V. Arrows indicate the sample addition and the washing with pure potassium phosphate buffer.

Further studies were conducted to detect circ-SHKBP1 in human plasma samples. A sample containing circRNA isolated from the analyzed plasma sample, as described in Section 4.8, was added to a measuring cell containing a clean buffer. Control experiments were conducted under the same conditions, but a sample of healthy volunteer plasma or a clean buffer was added to the cell. The results obtained in this experiment are shown in Figure 6.

Figure 6 shows that a significant change in the h-k-SOI-NW chip signal level was observed when circRNA samples isolated from the plasma of patients with diagnosed neoplasm of uncertain nature of the brain and central nervous system (sample No. 005, pink line) were added to the measuring cell. In the control experiments, when circRNA isolated from the blood plasma of a patient diagnosed with prostatic hyperplasia (sample No. 96, blue line) was used, the signal changes were practically not registered compared to the sensogram curves recorded in the working experiments. In addition, the signal did not change in that case, when a buffer solution was added to the measuring cell, which was prepared according to the same protocol as circRNA (Section 4.8). At the same time, the time required to detect the target circRNA was only 400 s (~7 min), while using classical clinical and laboratory methods, the analysis time is a few hours.

## 3. Discussion

As mentioned in the Introduction section, a method based on nanowire systems with sensitive elements is promising in the development of new highly sensitive methods for detecting glioma. The main problem in using such systems is the instability of the SOI-NW chip in liquid. To overcome this problem, a method of modifying SOI-NW chips using protective high-k layers was employed in the present study. Quality control of sensitive structures is essential while such layers are manufactured.

To monitor the quality of SOI-NW structures with an h-k layer with n-type conductivity, micro-Raman spectroscopy has been used. The Raman spectra for protective stacks made of a hafnium oxide nanolayer and with different numbers and thicknesses of aluminum oxide nanolayers were measured after heat treatment from 425 to 1000 °C. The stability of the signals from the monoclinic phase of hafnium dioxide was shown; the signals were decreasing as the amount of aluminum oxide in the stack was increasing. Significant differences were found in the elastic stresses of the silicon layers, which varied from tensile to compressive only for very thick (>200 nm) layers of aluminum oxide, as well as for sapphire substrates. In the UV spectra from the SOI layers and the silicon of the substrate with hafnium dioxide deposited on top, the arms of the single-phonon scattering signal were observed at 480–490 cm^−1^. Moreover, the same shoulders at 480–490 cm^−1^ were observed earlier on SOI NW structures with natural oxide on the silicon layer [34], but they were associated with defects in this layer. Presumably, these shoulders arise from the interlayer of silicon oxide, whose structure is similar to the natural oxide and the stretched sublayer of silicon at the same time. This question requires further research. 

Manufactured using optical lithography with gas plasma etching, h-k-SOI-NW chips with protective dielectric layers Al_2_O_3_ (2 nm) and HfO_2_ (8 nm) can be used for the label-free real-time detection of oDNAs with ~10^−16^ M concentration sensitivity. 

It should be noted that in a SOI sensor, changes in nanometer high-k layers of dielectrics are difficult to measure by Raman scattering in the backscattering geometry with a laser beam incidence perpendicular to the surface, due to the fact that even under ultraviolet excitation, a layer of hidden thermal silicon dioxide (playing a role in the same part of the spectrum as high-k dielectrics) is visible through an ultra-thin silicon layer. To increase the sensitivity in future studies, the following method is proposed: two sensor nanostrips are folded with the working side to each other using the technique of preparing samples for transmission electron microscopy of the cross-section (cross-section TEM or XTEM), then silicon dioxide is locally removed, and a laser beam is directed along the nanostrips bridge.

In the present work, studies were carried out to measure the response from the h-k-SOI-NW chip to the introduction of oDNA with a low concentration, in a buffer solution being a simplified model system. The concentration of 10^−16^ M was selected on the basis that the sub-femtomolar range of protein markers’ concentrations corresponds to the early stages of the disease [4]. To organize the biospecific registration of oDNA, the surface of the chip was covalently modified with complementary DNA probes. It was observed during the experiment that hybridization of oDNA with immobilized DNA probes resulted in a decrease in the conductivity of the h-k-SOI-NW chip n-type. The designed chips made it possible to register oDNA at a concentration of 1.1 × 10^−16^ M. Then, the chips were tested for the detection of circRNA in a multicomponent matrix. It has been shown that the h-k-SOI-NW chip allows recording of the changes in the level of circRNA in blood plasma samples obtained from patients diagnosed with neoplasm of uncertain nature of the brain and central nervous system. Taking into consideration the ultra-high sensitivity of the created h-k-SOI-NW chips, it can be concluded that in the future they could be employed to develop diagnostics of an early-stage glioma. This might be a big step forward in glioma detection based on liquid biopsy, which is painless for patients, less time-consuming, and facilitates the diagnosing processes both for doctors and patients. Moreover, such nanowire chips can be designed as a matrix with 10 or more channels for the simultaneous registration of the spectra of nucleic acid markers, which should increase the reliability of disease diagnosing.

## 4. Materials and Methods

### 4.1. Chemicals

Hydrofluoric acid (HF; «Reakhim», Moscow, Russia), isopropanol («Acros Organics», Geel, Belgium), 3-aminopropyltriethoxysilane (APTES) («Sigma-Aldrich», St.Louis, MA, USA), ethanol, toluene («Reakhim», Moscow, Russia), 3.3′-dithiobis (sulfosuccinimidyl propionate) (DTSSP) («Pierce», Appleton, WI, USA), deionized water was obtained with a Simplicity UV system («Millipore», Molsheim, France), potassium phosphate monobasic («Sigma-Aldrich», St. Louis, MA, USA).

### 4.2. Oligonucleotides

DNA oligonucleotides probe with the following sequence: 5′-$NH_{2}$-${\left(T\right)}_{10}$-GGGTGGGCAGGGAGGGTGGAGCGGGTTTGTTCCAAGTGCCCCAGCCTCTC; this sequence is complementary to that of the target oDNA (GAGAGGCTGGGGCACTTGGAACAAACCCGCTCCACCCTCCCTGCCCACCC), whose sequence corresponds to SHKBP1 circular RNA («Evrogen», Moscow, Russia).

### 4.3. Equipment

FlexAL equipment (Oxford Instruments, Abingdon, UK), Olympus BX41 microscope (Olympus Corp., Tokyo, Japan), Horiba Jobin-Yvon LabRam HR800 Raman spectrometer (HORIBA, Kyoto, Japan), ozonator UV Ozone Cleaner–ProCleaner™ Plus (Ossila Ltd., Sheffield, UK), Piezorray non-contact low-volume dispensing system (Perkin Elmer, Inc., Waltham, MA, USA), 10-channel data collection and storage system («Agama+»; JSC, Moscow, Russia), h-k-SOI-NW chip (Rzhanov Institute of Semiconductor Physics Siberian Branch of Russian Academy of Sciences (ISP SB RAS), https://www.isp.nsc.ru/en/, (accessed on 20 May 2021), Novosibirsk, Russia).

### 4.4. Fabrication of h-k-SOI-NW Chip

Chips were constructed by nanostructuring using NW structures (sensors) with n-type conductance. The NW structures have the following characteristics: the buried oxide (BOX) layer thickness is 300 nm; the cut-off silicon layer thickness is 30 nm; the thickness, the width and the length of each NW sensor are 30 µm, 3 nm and 10 µm, respectively. 

The dielectric layers were created in the form of structures HfO_2_, Al_2_O_3_/HfO_2_ and Al_2_O_3_/HfO_2_/Al_2_O_3_ on the surface of the silicon layer. Before applying the dielectric, the surface of the chips was cleaned in a peroxide-ammonia solution, and then in the Atomic Layer Deposition (ALD) box of the Flex Oxford machine for 2 min in NH_3_ plasma at a power of 500 W, under the pressure of 50 mTorr. The coating of the chips plasma-enhanced atomic layer deposition (PEALD) HfO_2_ 8 nm thick and PEALD Al_2_O_3_ 2 nm thick was carried out in a FlexAL machine, where the first precursor, the NW structures surface sorbed was tetramethylaminogaphnium (TMAN) or trimethylaluminium (TMA) at the temperature of 300 °C, and the second precursor was the ions from the oxygen plasma. All crystals with Al metallization were completely coated with a PEALD dielectric. The remote plasma source in the FlexAL machine provided an atomic oxygen concentration of 1013–1014 cm^−3^ in the reactor working area at a pressure of 15 mTorr, with a density of positively charged ions of ~109 cm^−3^. The table with the samples was under zero potential, which, combined with the above plasma parameters, had a “mild” stimulating effect on the heterogeneous oxidation reaction of TMAN or TMA without damaging NW structures. After applying the PEALD oxides, the crystals were annealed for 30 min in the forming gas (N_2_:H_2_ = 95:5) at the temperature of 425 °C and the pressure of 200 mTorr. The aluminum contact with n-type silicon shows a Schottky barrier with the height of up to 0.3 V, after PMA at 425 °C for 0.5 h. 

To acquire the micro-Raman spectra, a Horiba Jobin-Yvon LabRam HR800 Raman spectrometer was employed to recognize all layer compositions, stresses and phases, and structural defects. A built-in Olympus BX41 microscope allowed us to investigate sub-micrometer areas of field-effect transistors’ channels with the coating. Confocal optical scheme allowed us to achieve the maximum degree of detalization (which is necessary owing to small dimensions of the sensor elements), while maintaining a high speed of image acquisition upon excitation by beams (focused to 2 µm) of UV copper vapor laser (325 nm, 0.1 mW) or visible (Vis) argon gas (514.5 nm, 0.1 mW), or fiber-optic laser (532.1 nm, 0.1–1.0 mW). This has allowed us to prevent the heating of the coated nanosensors; confocal scheme enabled us to measure the properties of the dielectric layer directly on silicon film. Three laser wavelengths facilitated observed Raman peaks dispersion, as well as separation from luminescence peaks. Furthermore, the ultraviolet (UV) laser showed a high extinction coefficient, which is important for Raman signal excitation in the case of ultrathin films of silicon and cover dielectrics.

### 4.5. Modification of h-k-SOI-NW Chip Surface

To remove organic contaminants, first the surface of the sensor chip was prepared using aqueous isopropanol solution. Afterwards, to remove the native oxide formed during the storage of the chips, it was treated with a solution containing HF and ethanol. Then, to form hydroxyl groups on the surface of the NW, the chip was put into an ozonator. Next, the sensor chip was silanized in APTES vapors for 20 h at indoor temperature [35]. After the silanization, the surface of the chip was washed with ethanol and then dried.

### 4.6. Covalent Immobilization of Oligonucleotide Probes

To provide specificity of the detection of the target oDNA, the surface of the NW sensors was sensitized by covalent immobilization of oDNA probes complementing the target biomolecules (working NW sensors). For this purpose, firstly, the silanized NW sensor surface was activated first with DTSSP crosslinker. Secondly, 1 µM solution of oDNA probes in 50 mM potassium phosphate buffer were dispensed directly onto the surface of individual sensors using a Piezorray non-contact low-volume dispensing system. The volume of the oDNA solution, which was dispensed onto each nanowire sensor, made up ~0.6 nL. The oDNA solutions were incubated onto the sensor surface for 0.5 h at 15 °C, and after that they were washed away. The control NW sensors were not immobilized by oDNA probes.

### 4.7. Target oDNAs in Buffer Solutions Preparation

In 50 mM potassium phosphate buffer (pH 7.4) by serial ten-fold dilution with working buffer (1 mM potassium phosphate buffer, pH 7.4), the solutions of target oDNA with concentrations of 1.1 × 10^−16^ M were prepared from the initial 100 µM stock solution. The solutions were made immediately before the measurements.

### 4.8. Plasma Samples

Samples taken from patients diagnosed with neoplasm of uncertain nature of the brain and central nervous system were received from the Russian National Medical Research Center of Neurosurgery named after N. N. Burdenko (Moscow, Russia). The study was approved by an independent ethics committee based at the Russian National Medical Research Center of Neurosurgery named after N. N. Burdenko (protocol No. 12/2020, date of approval: 15 December 2020). Plasma samples from volunteers with prostatic hyperplasia diagnosis were received from the Institute of Urology and Reproductive Health (Sechenov University) (Protocol No. 10-19 of 17 July 2019) (Table 1). Written informed consents authorizing their participation in the study and the use of biological material were obtained from all donors. To ensure biological safety, all samples were deactivated before being used in the study.

To isolate miRNA from blood plasma samples, the miRCURY™-Biofluids RNA isolation kit (Exiqon A/S, Vedbaek, Denmark) was employed; the process of isolation was conducted in accordance with the manufacturer’s protocol. 

### 4.9. Electrical Measurements

The electronic measuring unit is designed for the concurrent registration of the signal from ten sensors (located on the chip), and for the real-time visualization of the signal on the personal computer screen during the experiment. The digitizing of the registered signal, the analysis and the visualization of the measurements in graphical form were carried out with a specially designed software («Agama+»; JSC, Moscow, Russia). During the measuring, the support of SOI structures was implemented as a control electrode (transistor gate). Real time rain-source current time dependencies I_ds_ (t) were recorded at V_g_ = 41 V and V_ds_ = 0.1 V. To increase the time stability of the NW sensors operation signal, an additional Pt electrode was submerged into the solution in the measuring cell [36].

## 5. Conclusions

In the present study, micro-Raman spectroscopy was used to control the manufacturing quality of sensor chips with a silicon-on-insulator nanowire structure (SOI). The results obtained indicated a good quality of the manufactured nanowires. The created chips showed good characteristics in terms of stability in liquid and buffer solution. Thus, it was shown that the h-k-SOI-NW chip allows the detection of oDNA (a synthetic analogue of circ-SHKBP1) in a buffer solution at a concentration of 1.1 × 10^−16^ M. To ensure biospecific detection of oDNA, the surface of the chip was covalently modified with DNA probes, which made it possible to register oDNA in real time. The results obtained in a model buffer solution for the successful registration of oDNA enabled the use of the h-k-SOI-NW chip for the highly sensitive detection of circRNA molecules associated with glioma development. The experiments conducted also resulted in the successful detection of circ-SHKBP1 in human blood plasma patients diagnosed with neoplasm of an uncertain nature of the brain and central nervous system. The approach proposed here can be applied in the development of novel highly sensitive methods of liquid biopsy of glioma, with undoubted advantages in identifying the disease with minimal inconvenience for patients. The developed method is low-cost, and the diagnosing process requires neither highly qualified personnel nor complex preparation procedures. Another important advantage is a real-time analysis (about 10 min) with femtomolar and sub-femtomolar sensitivity. This sensitivity is essential for the early diagnosis of cancer. It should be noted that this approach is promising, since it will allow the simultaneous use of several probes for several types of molecular targets due to a sensor matrix form of the chip.

## Figures and Tables

**Table 1:** Clinical and morphological characteristics of plasma samples.

Plasma Sample No.	Age	Sex	Diagnosis	Morphological Characteristic
005	54	Female	Neoplasm of an uncertain nature of the brain and central nervous system	Brain tissue with a marginal zone of glioma in one of the fragments, within the studied biopsy
96	70	Male	Prostatic hyperplasia	-

## Data Availability

Correspondence and requests for materials should be addressed to Y.D.I.
